# Editorial: Psychology and Policy

**DOI:** 10.3389/fpsyg.2017.00497

**Published:** 2017-04-11

**Authors:** Kai Ruggeri

**Affiliations:** Policy Research Group, Department of Psychology, University of CambridgeCambridge, UK

**Keywords:** behavioral sciences, policy, evidence-based policy, impact, social norms

Policies are population interventions aimed at guiding choices and behaviors to achieve a desired, repeated outcome. They involve sets of decisions, and standards used by a group when dealing with significant or common—or both—challenges. These scenarios typically require standardized approaches to ensure the most ideal outcome, whether quantifiable or ideological, or simply for consistency.

At some level, all policies are ideological, either in who is determined to be a population of interest or in what outcome is considered optimal. At their most tangible level, they are structured attempts to approach critical choices and best practice based on relevant data, though many policies appear by default and have no formal implementation. Such decisions will have substantial implications across a population, and should therefore be informed by a variety of sources while carried out by a plurality of stakeholders when possible, all by design.

At their most basic, policies may be unwritten codes of practice that result in consistent actions or series of steps when a group or individual faces a common choice or obstacle. Policies may be held by individuals, small groups, large groups, organizations, and diverse populations. They can be established by design or by default, as there are an infinite number of such potential tactics held by a complex overlap of individuals and arrays of groups.

Policies ultimately are actions (the *do*) of public, social, governmental and organizational leadership. They are not the rules or laws (the do *not*) for which individuals may be punished for violating, but instead are the strategies and approaches we take to achieve desired outcomes on population levels. While policies may stipulate regulations to be in place and vice-versa, this distinction is critical: policies seek to ensure sets of ideal behaviors and contingencies with an outcome in mind; laws merely set the rules of the game, not how to play it. To that effect, policies are most certainly precise actions aimed at carrying out a broader strategy. The implication is that not following leads to unwanted or suboptimal outcomes, which may result as a form of inherent, yet non-prescribed and self-determined punishment. Establishing these criteria in defining and translating evidence for policy is critical, given a significant lack of distinct terminology (Oliver et al., 2014). While they have a likely effect on many critical population outcomes, such as security, economic stability, and well-being, these are not always specified as the primary goal.

There is no doubt about the critical role psychologists play in making policies (Sunstein, 2015). With the seemingly rapid elevation to prominent influence across a number of domains, researchers in psychology must likewise expedite training in relevant competencies to ensure meaningful impacts for the next generation of psychological scientists. Psychology as a discipline has gone through rather distinct phases of focus on behaviorism followed by expanded interest in what is behind the behavior as well as what these implications have for individual and societal outcomes (Huppert, 2014). As such, psychological insights influencing major policies can be spotted in essentially every domain (Ruggeri, 2016).

Insights from psychology and behavioral sciences have long been vital in identifying and utilizing policy levers (Schneider and Ingram, 1990), which are actions taken based on data that can influence choices and behaviors leading to improved outcomes (Howlett et al., 2015). Parks et al. (2013) set out a comprehensive review of theories and empirical studies run by social psychologists that have direct implications not only for policies themselves, but for likely uptake across diverse populations. These newer tools relate heavily to work on social norms, which are not explicitly policies themselves but may feature heavily within them. Given the potential for policies to set such norms across large populations, there is considerable power in utilizing insights effectively, particularly in some of the most challenging areas of public interest (Parks, 2014).

## Research topic insights

In this volume, we covered a wide range of topics over the six manuscripts, which began with an active model of translation into practice from Werner-Seidler et al. from the Black Dog Institute, one of the world's leading mental health institutes. It also included a theoretical exploration of constructs from Mandel and Tetlock examining the value and role of academics in policy, most notably challenging the notion of neutrality. The next articles from Basso et al. and Flin et al. covered psychological insights from studies related to food-imitating products and obesity discrimination in the workplace, which are highly topical areas for researchers and policymakers alike. The latter article was covered heavily in the media around the time of its release. Finally, we included two educational studies, one covering the contextual factors associated with implementing positive psychology in schools from Ciarrochi et al. and another exploring implications of using multilevel models to assess standardized testing results in Brazil from Menezes et al. These studies demonstrate the need for psychological insights for not only innovative practice in education, but also for the methods used in analyzing them. These are particularly important given past arguments about lack of creativity in policymaking (Choi and Pritchard, 2003).

## The road ahead

The impact of psychology in policy—as well as the role of psychologists in policymaking—is well established. Going forward, there is a clear opportunity to present robust evaluation of where, how, and to what end this can be done to cover entire populations. As Mark Stokes, a neuroscientist at Oxford, once eloquently and diplomatically stated at a policy debate in 2013, “Policies may be evidence-based, but in the end, they are no more than untested interventions.” Responding empirically to such a statement would continue pushing psychological science to ever-rising prominence in contributing to effective policies. This would likely have the additional benefit of optimizing communication between science and policymakers. Advancing the role of psychological research would further address the current gap in theory that would unify existing behavioral insights for how policies can improve population outcomes. As this is an editorial, I would like to close by positing that most critically, the outcomes of greatest concern in policy are security, stability, and improved well-being across entire populations.

## Author contributions

The author confirms being the sole contributor of this work and approved it for publication.

### Conflict of interest statement

The author declares that the research was conducted in the absence of any commercial or financial relationships that could be construed as a potential conflict of interest.
